# Quantitative Analysis of Pancreatic Polypeptide Cell Distribution in the Human Pancreas

**DOI:** 10.1371/journal.pone.0055501

**Published:** 2013-01-31

**Authors:** Xiaojun Wang, Mark C. Zielinski, Ryosuke Misawa, Patrick Wen, Tian-Yuan Wang, Cheng-Zhang Wang, Piotr Witkowski, Manami Hara

**Affiliations:** 1 Department of Surgery, The University of Chicago, Chicago, Illinois, United States of America; 2 Institute of Hepatobiliary Surgery, Southwest Hospital, Third Military Medical University, Chongqing, China; 3 Department of Medicine, The University of Chicago, Chicago, Illinois, United States of America; University of Kentucky, United States of America

## Abstract

The pancreatic islet is mainly composed of beta-, alpha- and delta-cells with small numbers of pancreatic polypeptide (PP) and epsilon cells. It is known that there is a region in the head of the pancreas that is rich in PP-cells. In the present study, we examined the distribution of PP-cells, and assessed the influence of the PP-cell rich region to quantify the total islet mass. Pancreatic tissues were collected from donors with no history of diabetes or pancreatic diseases (n = 12). A stereological approach with a computer-assisted large-scale analysis of whole pancreatic sections was applied to quantify the entire distribution of endocrine cells within a given section. The initial whole pancreas analysis showed that a PP-cell rich region was largely restricted to the uncinate process with a clear boundary. The distinct distribution of PP-cells includes irregularly shaped clusters composed solely of PP-cells. Furthermore, in the PP-cell rich region, beta- and alpha-cell mass is significantly reduced compared to surrounding PP-cell poor regions. The results suggest that the analysis of the head region should distinguish the PP-cell rich region, which is best examined separately. This study presents an important implication for the regional selection and interpretation of the results.

## Introduction

While pivotal roles of insulin (secreted from beta-cells), glucagon (alpha-cells) and somatostatin (delta-cells) in glucose homeostasis have been well studied, the precise role of PP in the pancreas is largely unknown. In humans, inhibitory effects of PP on gallbladder contraction and pancreatic enzyme have been reported [1]. Animal studies suggest that PP may influence food intake, energy metabolism, and the expression of gastric ghrelin and hypothalamic peptides [2]–[5].

Beta-cells together with alpha- and delta-cells are organized into the islet of Langerhans, which is a functional unit. The distribution of these three major endocrine cells is similar, comprising of single cells and small clusters to large islets throughout the pancreas. A fewer number of epsilon-cells is found in the periphery of islets as well as scattered singlets in the pancreas [6]–[10]. However, the PP-cell distribution is distinct in the head region, where past studies reported that 55–90% of the islet cell volume in this location was represented by PP-cells [11]–[15]. Since the majority of recent studies to determine beta-cell/islet mass do not include PP-cells, and some studies rather focus on the PP-cell poor region (e.g. mid-portion or tail region of the human pancreas), we reason that it is important to determine to what extent the PP-cell rich region segregates and further examine the influence of inclusion or exclusion of a PP-cell rich region for the assessment of the total endocrine mass. Specifically, we aimed to test a hypothesis that the PP-rich segment in the head region should be studied independently for two reasons: (1) The overall contribution of PP-cells to islet mass is low, where only a restricted area in the head is PP-cell rich; and (2) the distinct islet morphology in the PP-cell rich region including irregularly shaped clusters composed solely of PP-cells.

Here we show that the restricted PP-cell rich area is largely in the uncinate process with some extension into the surrounding head region with a clear boundary from the PP-cell poor area. Furthermore, in the PP-cell rich region, islet cell composition significantly differs from the rest of the pancreas, accompanied by reduced beta- and alpha-cell mass. The present study suggests the importance of sampling tissues particularly in the head region, where the PP-rich region should be distinguished and analyzed independently.

## Materials and Methods

### Ethics Statement

The use of human tissues in the study was approved by the Institutional Review Board at the University of Chicago.

### Human pancreas specimens

Human pancreata were generously provided by the Gift of Hope Organ Procurement Organization in Chicago. Specimens were collected within 12 hours of cold ischemia. Clinical information about the donors (n = 12) is summarized in Table 1.

**Table 1:** Subject information.

Subject	Gender	Age	BMI	Cause of death
S1	M	20	21.2	Head trauma
S2	F	37	21.7	Cerebrovascular/stroke
S3	M	46	18.5	Anoxia/Cardiac arrest
S4	F	46	37.9	Anoxia
S5	M	50	23.2	Cerebrovascular/stroke
S6	M	50	32.0	Head trauma
S7	M	51	27.2	Cerebrovascular/stroke
S8	F	52	25.7	Cerebrovascular/stroke
S9	F	53	24.2	Cerebrovascular/stroke
S10	F	54	25.1	Cerebrovascular/stroke
S11	M	59	28.1	Cerebrovascular/stroke
S12	M	68	21.3	Head trauma

### Immunohistochemistry

Paraffin-embedded sections (5 µm) were stained with the following primary antibodies (all 1:500): polyclonal guinea pig anti-porcine insulin (DAKO, Carpinteria, CA), mouse monoclonal anti-human glucagon (Sigma-Aldrich, St. Louis, MO), polyclonal rabbit anti-pancreatic polypeptide (DAKO), polyclonal goat anti-somatostatin (Santa Cruz, Santa Cruz, CA) and DAPI (Invitrogen, Carlsbad, CA). The primary antibodies were detected using a combination of DyLight 488, 549, and 649-conjugated secondary antibodies (1:200, Jackson ImmunoResearch Laboratory, West Grove, PA).

### Image capture and quantification

Microscopic images were taken with an Olympus IX8 DSU spinning disk confocal microscope (Melville, NY) with imaging software StereoInvestigator (SI, MicroBrightField, Williston, VT). A modified method of “virtual slice capture” was used [16]–[18]. Briefly, the SI controls a XYZ-motorized stage and acquires consecutive images, which creates a high-resolution montage composed of images obtained from multiple microscopic fields of view. The entire tissue section was captured as “a virtual slice” using a 10x objective. Each virtual slice taken at four fluorescent channels were further merged into one composite. Quantification of cellular composition (i.e. each area of PP-, beta-, alpha-, and delta-cell populations, and islet area by automated contouring of each islet) was carried out using a macro custom-written for Fiji/ImageJ (http://rsbweb.nih.gov/ij/). MATLAB (MathWorks, Natick, MA) was used for mathematical analyses.

### Statistical analysis

Data are expressed as mean ± SEM. Statistical analyses were performed using paired Student's *t* test. Differences were considered to be significant at *P*<0.05.

## Results

### Whole pancreas analysis showed the restricted PP-cell rich area to the uncinate process with some extension to the surrounding head region

**Figure 1:**
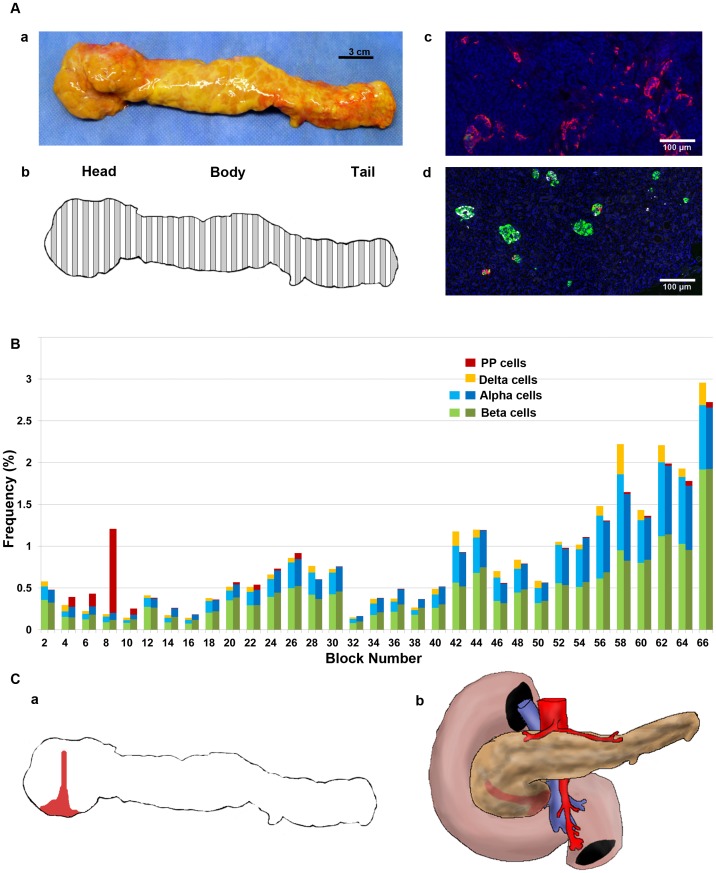
Whole pancreas analysis of the PP-cell distribution. **A**: **a**. A pancreas from a 50-yr old male; S5). **b**. Collection scheme. Specimen divided into 66 consecutive sections, with preparation alternating between fresh-frozen (white) and paraffin embedding (grey). The latter set of tissue blocks was used in the present study. **c**. Representative view of the PP-cell rich region with irregularly shaped clusters of PP-cells. Immunostained for PP (red), insulin (green), glucagon (white) and nuclei (blue). **d**. PP-cell poor area. **B**: Regional distribution of PP-, beta-, and alpha-cell mass. **C**: **a**. Restricted PP-cell rich area illustrated in red. **b**. Deduced PP-cell rich region *in vivo*.

We first examined the extent of the PP-cell rich region in a whole pancreas from a 50-yr old male (S5; Fig.1A.a). The pancreas was divided into 66 consecutive tissue blocks and paraffin-embedded sections were used in this study (Fig.1A.b). The PP-cell rich area in the head region exhibited the distinct morphology of irregularly shaped PP-cell clusters as shown in Fig.1A.c, whereas in other regions throughout the pancreas, a small number of PP-cells were scattered or found in islets (Fig.1A.d). Regional distribution of the total endocrine cell area per tissue section was examined first for the three major endocrine cells (beta-, alpha- and delta-cells). Then adjacent sections in each block were stained for PP with reference to beta- and alpha-cells. Note that currently quadruple staining including nucleus staining (e.g. DAPI) is the limit, since a far-red secondary antibody (e.g. Cy7) does not provide consistent staining. Differences between two adjacent sections in the total beta- and alpha-cell area were 0.04±0.004% and 0.04±0.005%, respectively. The result is plotted from the head-body-tail region, which shows a narrowly restricted PP-cell rich area that does not cover the entire head region (Fig.1B). A deduced PP-cell rich region in the pancreas specimen as well as *in vivo* is illustrated in Fig.1C.a and b, respectively.

**Figure 2:**
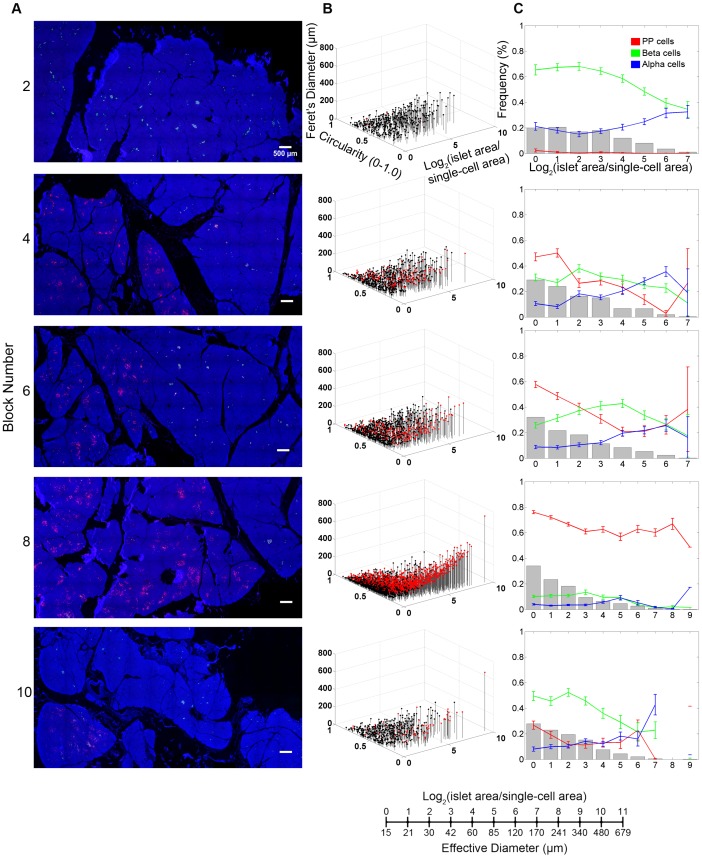
Detailed distribution of the PP-rich region in the head of the pancreas. **A**: Virtual slice views of blocks in the head region. Immunostained for PP (red), insulin (green), glucagon (white) and nuclei (blue). All in the same scale. **B**: 3D visualization of islet size (area) and shape (circularity and Feret's diameter) distribution. Each dot represents a single islet/cluster. PP-cell containing clusters are shown in red. **C**: Quantitative analysis of individual islet size distribution and cellular composition. Relative frequency of islet size (gray bar) and ratios of beta (green), alpha (blue), and PP (red) cells within islets are plotted against islet size; means ± SEM.

Further analyses of the PP-cell rich region on the islet size distribution and cellular composition depict changes over the transverse transition through the head region (Fig.2). Representative views of each block are shown in Fig.2A. In Panel B, islet size and shape distribution are visualized three-dimensionally. Here, in addition to each islet area, we also measured circularity (which reports the roundness of a structure where 1.0 depicts a perfect circle) and Feret's diameter (the longest distance within a structure). Each dot represents a single islet/cluster and PP-cell containing structures are indicated in red. For example, large irregularly shaped PP-cell clusters fall into a location of higher values of area and Feret's diameter accompanied by lower circularity. Note that islet area/size is presented as a logarithmic scale considering the high number of small islets and the low number of large islets. The conversion between logarithmic islet area and effective diameter (µm) is provided. Quantitative analysis of islet size distribution and cellular composition is shown in Panel C. As highlighted in the Block #8, the PP-cell rich region exhibits a distinct endocrine cell composition from the surrounding regions.

### Reduced beta- and alpha-cell mass in the PP-cell rich region

The regional difference of PP-cell distribution in the head, body and tail regions was examined in multiple specimens (n = 11). Sections from each region were collected as follows: head from a region near the duodenum; body right after the neck; and tail at the end of pancreas. PP-cell distribution in the body and tail regions was consistently low (body: 0.02±0.01%; tail: 0.02±0.008%) in all specimens. In the head region, the fraction of the PP-cell rich area varied among specimens, which may suggest individual differences in the extent of segregation (see Discussion).

**Figure 3:**
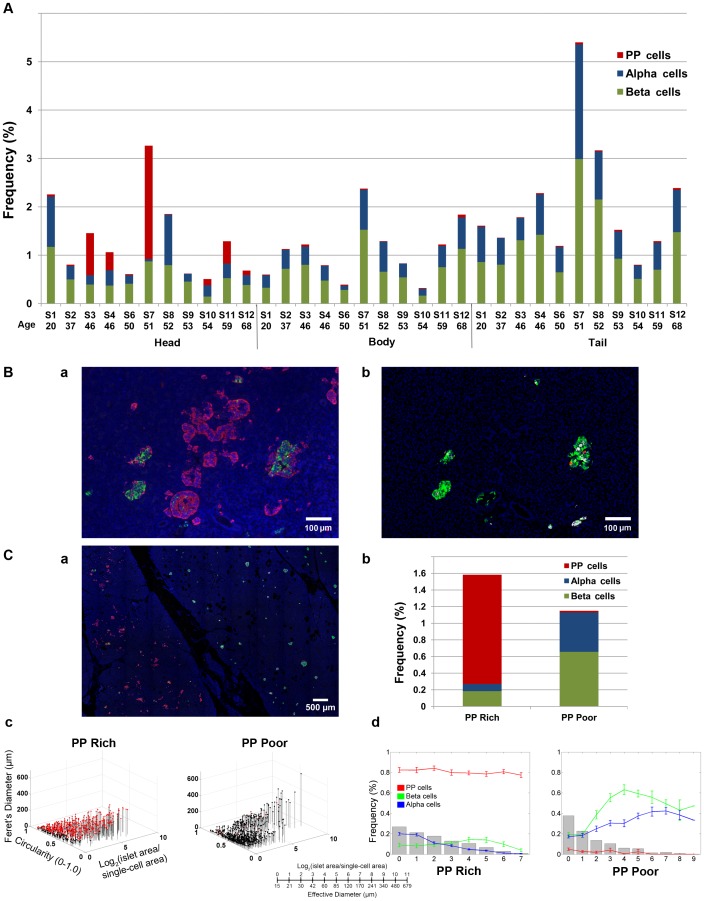
Analysis on multiple specimens. **A**: Inter-specimen comparison of endocrine cell mass in the head, body and tail region. **B**: **a**. Representative view of islets in the PP-cell rich area (PP in red, insulin in green, glucagon in white and nuclei in blue). **b**. Adjacent section immunostained for insulin (green), glucagon (red), somatostatin (white) and nuclei (blue). **C**: Intraspecimen comparison of the PP-cell rich and poor regions. **a**. A clear boundary between the PP-cell rich (area in the left) and poor region (right). **b**. Total endocrine cell area in each region. **c**. 3D plot of individual islet/cluster with PP-cell containing clusters in red. PP-cell rich (left) and poor area (right). **d**. Islet size distribution and cellular composition. PP-cell rich (left) and poor area (right).

The PP-cell rich region contained a large number of irregularly shaped structures as well as islets with PP-cells in the periphery (Fig.3B.a), which are masked when only three major hormones are stained (adjacent section in Fig.3B.b). To assess the influence of inclusion of the PP-cell rich region on the total endocrine cell mass, intraspecimen comparison of the PP-cell rich and poor regions was carried out (Fig.3C.a; S4). In the PP-cell rich region, with a significant amount of PP-cells, both beta- and alpha-cell areas are significantly reduced compared to the PP-cell poor region (Fig.3C.b). The average differences in endocrine cell areas in specimens that contained both areas within a section (S3, S4 and S10) between the PP-cell rich and poor areas were PP-cells 1.2±0.08% and 0.01±0.004%; beta-cells 0.3±0.07% and 0.7±0.06%; and alpha-cells 0.08±0.03% and 0.5±0.01% (all *P*<0.05), respectively. Note that a specimen from S6 only contained a PP-cell rich region. The analysis on PP-cell rich and poor areas in the same section (S4; shown in Fig.3C.c and d) highlights the differences in the distribution of islets/clusters and cellular composition.

## Discussion

The large size of the human pancreas requires a stereological approach to quantitatively analyze endocrine cell mass. Commonly used methods include the measurement of multiple regions out of a single section or a certain number of islets per section, and the average of the triplicate (or more) is considered to represent the total endocrine cell area. Additional conversion of the 2D area value to 3D mass using pancreas weight may further confound accuracy. We have shown a possible sampling bias by selecting islet-rich areas of a section, while a large number of endocrine cells scatter as singlets and small clusters and a relatively smaller number of large islets consisting of several thousands of endocrine cells unevenly distributed in the pancreas [16]. Moreover, the cellular composition and architecture of the human islet is size-dependent that the intermingled beta- and non-beta-cells are only observed in large islets [19]–[21], which is not an intrinsic characteristic of human islets, but is also observed in mice under insulin resistance such as pregnancy, obesity, diabetes and inflammation [19], [22]. In order to minimize a sampling bias with a practical stereological approach, we have developed a computer-assisted large-scale image analysis of the entire section that provides information on every endocrine cell mass (from singlets to large islets) such as area, shape, cellular composition, and islet architecture (i.e. coordinates of each endocrine cell within a given islet) [16], [20], [21]. The whole pancreas analysis in this report provides an example of how endocrine cell mass changes regionally in an individual pancreas. It is therefore important for an interspecimen comparison to identify precise locations where specimens are collected.

In the present study, we have shown that the PP-rich region is more narrowly restricted to the uncinate process with some extension into the surrounding head region than previously reported [11]–[15]. Previous studies basically took a similar approach as ours using whole pancreata divided into 8 parts (4 parts from the head region) and analyzed one block per part. Rigorous analyses were performed using point-counting morphometry. A PP-cell rich area was found in the head region with considerable variability in all studies overall from 0% to ∼90%, which is consistent with the results of the present study. However in these studies, the PP-cell rich area in the head region was compared to that of the body and tail regions (which are referred to the PP-cell poor region or the alpha-cell rich region). Such comparison may lead to an interpretation that the head region is largely PP-cell rich. Specifically, we have shown that the PP-cell rich and poor areas coexist in the head region with a clear boundary. PP-cell distribution in a PP-cell poor area in the head region is similar to the rest of the pancreas.

The uncinate process is a portion of the pancreas posterior to the superior mesenteric vein and medial to the head of the pancreas. While the uncinate process has received little attention in the field of endocrinology, clinical studies on the diagnosis of pancreatic cancer report the importance of understanding its physiological properties and normal morphological variations. Jacobsson et al showed a common false-positive uptake of a tracer ^68^Ga-DOTA-TOC by the uncinate process (suggested due to the dense expression of somatostatin receptors in PP-cells) [23]. Chandra et al reported common variations in the morphology of the head and uncinate process arisen from early development that can be misdiagnosed as pancreatic tumor [24]. During development, the uncinate process forms from the ventral bud and the intestine undergoes ∼270° anticlockwise rotation, then it fuses with the dorsal bud. It is conceived that the failure of the full rotation results in abnormal growth and malpositioning of the dorsal or ventral bud prior to fusion, leading to the enlargement of the head associated with hypoplasia or aplasia of the uncinate process. The frequent physiological variations of the size and positioning of the uncinate process may explain the random inclusion of the PP-rich region in specimens from the head region, as demonstrated in the present study. Interestingly, the majority of pancreatic cancer is found in the head region [25]–[29], which receives a blood supply (superior pancreaticoduoodenal artery) different from the body and tail region (splenic artery). It is possible that the head region has distinct properties, anatomically and/or developmentally, that could lead this region being more prone to be affected in disease states. In fact, diabetes increases the risk of developing pancreatic cancer ∼2-fold [30], [31]. Taken together, pathological changes in the head region may hold a key to understanding pancreatic disease states, and thus should not be excluded.

It is of interest to examine the functional properties of these PP-rich islets. High plasma concentrations of PP have been reported in patients with endocrine pancreatic tumors [32], [33] as well as in diabetic patients [34], [35]. A recent study by Kahleova et al. showed an association of decrease in PP secretion with improvement in beta-cell function after diet-induced weight loss in subjects with type 2 diabetes [36].

In summary, the present study has demonstrated possible sources of sampling biases that have an important implication for accurate measurement of endocrine cell mass in the human pancreas. Our current effort focuses on the whole pancreas analysis of individuals in a wide range of ages as well as under disease conditions such as obesity and diabetes. Such studies should lay a baseline to establish standardized sampling and interpretation of results throughout the scientific community.
